# A survey of the East Palaearctic Lycosidae (Araneae). 9. Genus *Xerolycosa* Dahl, 1908 (Evippinae)

**DOI:** 10.3897/zookeys.119.1706

**Published:** 2011-07-15

**Authors:** Yuri M. Marusik, Mykola M. Kovblyuk, Seppo Koponen

**Affiliations:** 1 Institute for Biological Problems of the North, Portovaya Str. 18, Magadan 685000 Russia; 2 Zoological Department, V.I. Vernadsky Taurida National University, Yaltinskaya Str. 4, Simferopol 95007, Crimea, Ukraine; 3 Zoological Museum, University of Turku, FI-20014 Turku Finland

**Keywords:** Wolf spider, Asia, new combination, new synonymy

## Abstract

Three species of *Xerolycosa*: *Xerolycosa nemoralis* (Westring, 1861), *Xerolycosa miniata* (C.L. Koch, 1834) and *Xerolycosa mongolica* (Schenkel, 1963), occurring in the Palaearctic Region are surveyed, illustrated and redescribed. *Arctosa mongolica* Schenkel, 1963 is removed from synonymy with *Xerolycosa nemoralis* and transferred to *Xerolycosa*, and the new combination *Xerolycosa mongolica* (Schenkel, 1963) **comb. n.** is established. One new synonymy, *Xerolycosa undulata* Chen, Song et Kim, 1998 **syn.** **n.** from Heilongjiang = *Xerolycosa mongolica* (Schenkel, 1963), is proposed. In addition, one more new combination is established, *Trochosa pelengena* (Roewer, 1960) **comb. n**., ex *Xerolycosa*.

## Introduction

This paper is the first in a series of reviews of the Palaearctic Evippinae Zyuzin 1985. Evippinae is a relatively small subfamily of wolf spiders restricted to Africa and the Palaearctic Region. Only four species belonging to two genera have been recorded from Europe, *Xerolycosa nemoralis* (Westring, 1861) and *Xerolycosa miniata* (C.L. Koch, 1834) (both occur throughout Europe), *Evippa eltonica* Dunin, 1994 (easternmost Europe, only a few dozen kms from Asia) (Helsdingen 2010) and “*Evippa*” *apsheronica* Marusik, Guseinov & Koponen, 2003 (Ponomarjov and Tsvetkov 2004; Kovblyuk 2007). Most Evippinae species in the Palaearctic Region have been reported and described from Central Asia and China (cf. Platnick 2011). *Xerolycosa* Dahl, 1908 was assigned to the Evippinae by Zyuzin (1985). It is the most widespread genus in the subfamily, ranging from the Iberian Peninsula to Kamchatka. The genus currently includes five species (Platnick 2011), three of which are restricted to the Palaearctic Region and two occur in the Afrotropical Region. The purpose of this paper is to provide a review of this small genus.

## Material and methods

Specimens were photographed using either a JEOL JSM-5200 scanning electron microscope or an Olympus Camedia E-520 camera attached to an Olympus SZX16 stereomicroscope at the Zoological Museum, University of Turku. Digital images were montaged using a “CombineZM” image stacking software. Photographs were taken in dishes of different sizes with paraffin at the bottom. Different sized holes were made in the bottom to keep the specimens in the correct position. Figures had been made previously and in some cases we were unable to generate scale bars for the digital photographs. All measurements are given in mm. Drawings we made either by using a grid method with a MBS-9 stereomicroscope or a Leitz stereomicroscope with a camera lucida. The bleached epigyne of the holotype female was temporarily coloured with Chlorazol Black. Epigynes were macerated using KOH solution. In the tables of leg spination, apical and dorsal spine data are omitted.

Terminology of the copulatory organs follows (Zyuzin (1985, 1993).

Abbreviations used in the text: AME, ALE, PME, PLE – anterior median, anterior lateral, posterior median and posterior lateral eyes respectively; pv – proventral; rv – retroventral; v – ventral; p – prolateral; r – retrolateral.

**Acronyms:**

IBPNInstitute for Biological Problems of the North, Magadan, Russia

MNHNMuséum National d'Histoire Naturelle, Paris, France

SMFMSenckenberg Museum, Frankfurt am Main, Germany

SZMNSiberian Zoological Museum RAS, Novosibirsk, Russia

TNUZoology Department, Taurida National University, Simferopol, Ukraine

ZISPZoological Institute, St.-Petersburg, Russia

ZMMUZoological Museum of the Moscow State University, Russia

ZMUTZoological Museum, University of Turku, Finland

## Taxonomic survey

### Xerolycosa

Dahl, 1908

http://species-id.net/wiki/Xerolycosa

Xerolycosa Dahl, 1908: 361. Type species: *Lycosa nemoralis* Westring, 1861.
Saitocosa Roewer, 1960: 889. Type species: *Tarentula flavitibia* Saito, 1934.

#### Diagnosis.

Members of this genus can be easily separated from otherEvippinae genera by the fewer number of ventral tibial spines on leg I (3pv & 2rv, or 2–2v), carapace lacking transverse depression (present in *Evippa* Simon, 1882) and lack of pseudo-articulation of tarsi (Fig. 13). *Xerolycosa* can be differentiated by the shape of their copulatory organs. Females have a short droplet-shaped septum (about as long as wide), while in *Evippa* the septum is long and has a well developed septal stem. The male palp in *Xerolycosa* has a shorter course of the seminal duct and a shorter embolus, which is only partly hidden by the tegulum.

#### Description.

Medium-sized (5.5–7.5) dark coloured or spotty lycosids. Carapace spotty or dark coloured with lighter median band and two lateral stripes. Cephalic region not elevated. Chelicerae with 3 promarginal and 2 retromarginal teeth. Inner side of chelicerae with a kind of stridulatory file (Fig. 12). Femora with 3 dorsal spines, tibia and metatarsus with 2 dorsal spines, sometimes poorly developed, tibia and metatarsi with four or five ventral spines (3pv-2rv or 2–2v). Tarsi without transverse furrow.

Male palp: cymbium with several apical spines; tegular apophysis shifted retrolaterally, with bill-like extension directed ventrally. Palea absent, embolus forming almost a circle, only partly hidden by tegulum. Epigyne: fovea (depression) absent, septum droplet-shaped, covered with hairs, almost as wide as high; stem short. Weakly sclerotized parts of epigyne are referred to here as windows (*Wi*).

#### Comments.

*Saitocosa* was synonymised with *Xerolycosa* by Yaginuma (1986: p. 169) through synonymisation of the type species *Tarentula flavitibia* Saito, 1934 with *Xerolycosa nemoralis.*

Dahl (1908) described *Xerolycosa* and placed only two species in this genus: *Xerolycosa nemoralis* and *Xerolycosa miniata*. No type species was selected. It is not clear who selected *Xerolycosa nemoralis* as the type species. The first clear indication we found was in Roewer's catalogue (Roewer 1954: p. 309). The same species was indicated as the generotype in Roewer's revision of Lycosidae (Roewer 1959: p. 893) and in Bonnet's catalogue (1959: p. 4836).

In Platnick's catalogue (2011) five species are listed under *Xerolycosa*: *Xerolycosa miniata* (C.L. Koch, 1834), *Xerolycosa nemoralis* (Westring, 1861), *Xerolycosa pelengena* Roewer, 1960,*Xerolycosa sansibarina* Roewer, 1960 and *Xerolycosa undulata* Chen, Song et Kim, 1998. Roewer's species are known from Africa (Congo and Zanzibar). Judging from the figures, *Xerolycosa pelengena* is a member of Trochosini, due to its carapace pattern (two dark longitudinal stripes within the median band, just behind the PLE) and epigyne (anchor-shaped septum, and triangle-shaped hoods of the apical pocket) and seems to belong to *Trochosa*. Therefore, we propose the new combination: *Trochosa pelengena* (Roewer, 1960) comb. n. *Xerolycosa sansibarina*, known from the male sex only, has a carapace and abdominal pattern very different from Evippinae species, and the palp has a distinctly different conformation, typical for the Lycosinae (tegular apophysis stretching horizontally, tip of embolus visible and resting horizontally in a tegular depression). However, we refrain from suggesting a new combination because its generic affinities are currently unclear.

Because of the burrowing behaviour in *Xerolycosa mongolica* (Schenkel, 1963), believed to be absent in the other species, we first followed A.A. Zyuzin's (personal communication) opinion that it may belong to a separate genus. However, females of *Xerolycosa nemoralis* are known to excavate shallow depressions in soil (Smola 2007). In addition to behaviour, *Xerolycosa mongolica* has widely spaced posterior median eyes (one diameter apart) in contrast to the type species, *Xerolycosa nemoralis*, and *Xerolycosa miniata* (less than one diameter apart). Study of the male palp and the leg spination revealed no differences between *Xerolycosa mongolica* and the other species.

## Species separation

*Xerolycosa* species can be distinguished by the shape of the copulatory organs. In addition *Xerolycosa mongolica* can be recognized by the variegated (spotty) pattern of the carapace and abdomen, and by lacking a light median band. The spination of leg I may help to distinguish males of *Xerolycosa mongolica*, and females of all species.

The male palps in all three species are rather similar in general appearance. The species can be relatively easy recognized in retrolateral view by the profile of the tegular apophysis (Figs 22–24, 25, 27, 29) and by the shape of the embolic region following dissection, notably the course and length of the embolus, and the seminal duct position (Figs 26, 28, 30). The males of *Xerolycosa miniata* and *Xerolycosa nemoralis* have the same spination pattern on leg I (Table 1), but the females have different leg spine formulae (Table 2). The epigynes in the three species are very similar and can be distinguished by the shape of the septum and the “windows” (Figs 31, 33, 35, 37, 39, 41). Additional differences can be found in the spermathecae (Figs 32, 36, 40, 34, 38, 42).

**Table 1.** Chaetotaxy of leg I in *Xerolycosa* males.

Species	Segments of leg I			
	femur	patella	tibia	metatarsus
miniata	2p+2r	1p+1r	1p+2r+3-2v	2p+1r+2-2v
mongolica	1 or 2p+2r	1p	0 or 1p+2-2v	1p+2-2v
nemoralis	2p+2r	1p+1r	1p+2r+3-2v	2p+1r+2-2v

**Table 2.** Chaetotaxy of leg I in *Xerolycosa* females

Species	Segments of leg I			
	femur	patella	tibia	metatarsus
miniata	2p	0	1p+2-2v	2p+2-2v
mongolica	2p	0	0 or 1p+3-2v	2p+2-2v
nemoralis	2p+2r	1p	1p+3-2v	2p+2-2v

### Key to the Palaearctic *Xerolycosa* species

**Table:** 

1	Carapace with wide whitish median band	2
–	Carapace without whitish median band	*Xerolycosa mongolica*
2	Tegular apophysis with well developed ridge, and lower part as high as upper part (Fig. 25), free part of embolus bent (Fig. 26), epigynal windows wider than high (Figs 39, 42)	*Xerolycosa nemoralis*
–	Tegular apophysis has no developed ridge and lower part is higher than upper part (Fig. 27), free part of embolus gradually rounded, epigynal windows droplet-shaped, higher than wide (Figs 31, 33)	*Xerolycosa miniata*

## Species survey

### Xerolycosa miniata

(C.L. Koch, 1834)

http://species-id.net/wiki/Xerolycosa_miniata

Figs 6–7 22 27–28 31–34

Lycosa miniata C.L. Koch, 1834: 123, pl. 13–14 (♂♀).
Xerolycosa miniata : Dahl 1908: 361, 364, f. 58 (♂♀).
Xerolycosa miniata : Holm 1947: 24, pl. 4, f. 34–35, pl. 10, f. 26 (♂♀).
Xerolycosa miniata : Roberts 1985: 142, f. 61b (♂♀).
Xerolycosa miniata : Roberts 1995: 223, f. (♂♀).
Xerolycosa miniata : Roberts 1998: 237, f. (♂♀).
Xerolycosa miniata : Almquist 2005: 251, f. 245a-f (♂♀).

For a complete list of references see Platnick (2011).

#### Material examined.

**FINLAND** (selected records from different parts of the range): 15♂♀ (ZMUT), Nauvo Seili (60°15'N, 21°58'E), sandy sea shore, 16.05.-11.08.1974 (R. Mannila); 1♂ 1♀ (ZMUT), Virolahti Siikasaari (60°28'N, 27°35'E), sandy sea shore, 07.05.-13.09.1970 (S. Kännö); 17♂♀ (ZMUT), Pori Yyteri (61°33'N, 21°32'E), among *Empetrum* in sand dune, 14.07.1968 (P.T. Lehtinen); 1♀ (ZMUT), Hailuoto Marjaniemi (65°02'N, 24°36'E), *Elymus* sandy shore, 12.7.1973 (P.T. Lehtinen). **RUSSIA**:Adygeya: 1♂ 1♀ (TNU-2657/18), Caucasian State Reserve, 12 km SE kordon Guzeripl, Pastbishche Abago Mt. Range (43°53'N, 40°12'E 43°56'N, 40°16'E, 1727–2010 m a.s.l.), 18–23.08.2009 (M.M. Kovblyuk). Tuva: 13♂ 11♀ (IBPN), Uyuk River mouth, 52°04'N, 94°22'E, 600–700 m, 3–5.06.1995 (Y.M. Marusik). UKRAINE: Crimea: 1♀ (TNU-2187/1), Simferopol Distr., Kesslers' Forest, 8.08.2000 (M.M. Kovblyuk).

#### Diagnosis.

The species differs distinctly from *Xerolycosa mongolica* by the carapace pattern, having a light longitudinal band and stripes. Males can be distinguished from those of *Xerolycosa nemoralis* by the shorter seminal duct, a bent free part of the embolus and a bent tip, a rounded (not pointed) process of the tegular apophysis, basal part higher than apical (equal in *Xerolycosa nemoralis*), and the lack of a tegular ridge. Females can be distinguished by the proportions of the epigyne (windows longer than wide, whereas in *Xerolycosa nemoralis* they are wider than long).

#### Description.

##### Male.

Total length 5.0 (4.7–6.2). Carapace: 2.85 (2.52–3.09) long, 2.1 (1.79–2.22) wide. Carapace length/femur IV ratio 1.2. Habitus and pattern as in Fig. 6; carapace with wide white median band and marginal light stripes.

Palp as in Figs 22, 27–28, cymbial spines poorly distinct, upper part of tegular apophysis with claw-like outgrowth; embolus relatively thin, following an oval course, tip modified.

**Table:** Length of leg segments:

	femur	patella	tibia	metatarsus	tarsus	Total
I	1.95	0.85	1.5	1.6	1.13	7.03
II	1.8	0.8	1.35	1.55	1.15	6.65
III	1.85	0.8	1.3	1.85	1.05	6.85
IV	2.38	0.95	1.85	2.8	1.38	9.35

**Table:** Spination of legs:

	femur	patella	tibia	metatarsus
I	2p+2r	1p+1r	1p+2r+3-2v	2p+1r+2-2v
II	2p+2r	1p	2p+2r+2-2v	2p+1r+2-2v
III	2p+2r	1p+1r	2p+2r+2-2v	2p+2r+2-2v
IV	2p+1r	1p+1r	2p+2r+2-2v	2p+2r+3-2v

##### Female.

Total length 7.0 (4.8–7.4). Carapace: 3.0 (2.46–3.28) long, 2.1 (I. 71–2.42) wide. Carapace length/femur IV ratio 1.25. Habitus and pattern as in Fig. 7; pattern on carapace same as in male but with less distinct lateral light stripes.

Epigyne as in Figs 31–34, sides of stem rounded, windows shaped like inverted droplets, their upper margins almost horizontal.

**Table:** Length of leg segments:

	femur	patella	tibia	metatarsus	tarsus	Total
I	1.75	0.93	1.35	1.35	1.05	6.43
II	1.75	0.9	1.25	1.38	1.03	6.3
III	1.8	0.88	1.2	1.7	1.03	6.6
IV	2.4	1.05	1.85	2.7	1.3	9.3

**Table:** Spination of legs:

	femur	patella	tibia	metatarsus
I	2p	0	1p+2-2v	2p+2-2v
II	2p	1p	1p+2-2v	2p+2-2v
III	2p+2r	1p+1r	2p+2r+2-2v	2p+2r+2-2v
IV	2p+1r	1p+1r	2p+1r+2-2v	2p+2r+3-2v

#### Distribution.

*Xerolycosa miniata* has a Euro-Mongolian boreo-nemoral range (Marusik et al. 2000) and is known from Portugal to Tuva, north to central Finland and north Ural, and south to Azerbaijan and north-western Mongolia.

### Xerolycosa mongolica

(Schenkel, 1963) comb. n.

http://species-id.net/wiki/Xerolycosa_mongolica

**Figures 1–7.**
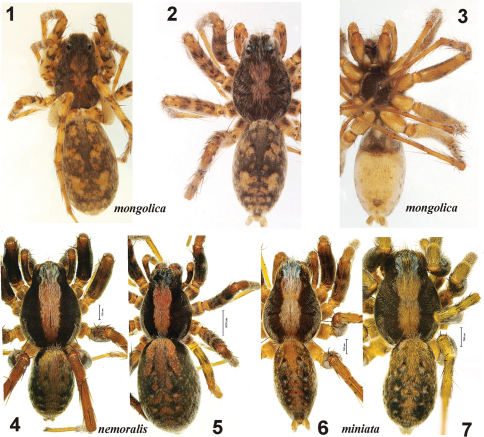
General appearance of *Xerolycosa mongolica* **1–3** *Xerolycosa nemoralis* **4–5** and *Xerolycosa miniata* **6–7** **1, 5, 7** female, dorsal **2, 4, 6** male, dorsal **3** male, ventral.

Figs 1–3 10 18–21 23a-b 29–30 35–38.

Arctosa mongolica Schenkel, 1963: 353, f. 204a-c (♀).
Xerolycosa nemoralis : Yu & Song 1988: 118 (incorrect synonymy).
“Xerolycosa” mongolica : Logunov, Marusik & Koponen 1998: 139.
“Xerolycosa” mongolica : Marusik, Logunov & Koponen 2000: 87.
Xerolycosa undulata Chen, Song & Kim, 1998: 71, f. 7–12 (♂). syn. n.
Xerolycosa undulata : Song, Zhu & Chen 1999: 346, f. 202J (♂).

#### Material examined.

Holotype ♀ (MNHN) “Urga-Tsitsikar, Chaffanjon” [1896] (can refer either to Mongolia or China). **RUSSIA**, Tuva**:** 17♂ 2♀ (IBPN & ZMUT), SE Tuva, Erzin Town environs, 50°14'N, 95°09'E, 1165 m, dry steppe, 9.06.1995 (Y.M. Marusik & S. Koponen); 13♂ 1♀ (SZMN), SE Tuva, Erzin environs, 50°14'N, 95°09'E, 1165 m, *Artemisia-Stipa* steppe, 9.06.1995 (D.V. Logunov); 4♂ (ZMMU), SE Tuva, Tes-Khem Valley, 50°19'N, 95°01'E, 10.06.1995 (Y.M. Marusik); 3♂ (ZMMU), environs of Kyzyl, *Nanophyton erinaceus* semidesert steppe, 6.06.1995 (Y.M. Marusik).

#### Notes.

The holotype female of *Xerolycosa mongolica* is very pale and the pattern is not visible. The figure in Schenkel (1963), however, corresponds well with the pattern observed in Tuvan specimens. When Yu and Song (1988) synonymized *Arctosa mongolica* and *Xerolycosa nemoralis* they mentioned that the type of *Arctosa mongolica* had been studied. The general appearance of the epigynes in the two species is not similar. The epigynal septum and the windows are more similar to those in *Xerolycosa miniata* (cf. Figs 31, 33, 35 and 37).

*Xerolycosa undulata* was described on the basis of the holotype male from Heilongjiang, not far from Tsitsikar. According to the text (Chen et al. 1999), the type was deposited in the Institute of Zoology in Beijing. However, the type was not found in the collections (Li, personal communication). Comparison of our figures of the male palp of *Xerolycosa mongolica* and figures of *Xerolycosa undulata* provided by Chen et al. (1998) leaves no doubts that these two names should be synonymized. It is worth mentioning, that when *Xerolycosa undulata* was described the male of *Xerolycosa mongolica* was unknown.

#### Diagnosis.

 *Xerolycosa mongolica* differs distinctly from its congeners by its spotty pattern and lack of longitudinal bands or stripes on the carapace, widely spaced anterior median eyes (more than one diameter of AME), long filiform embolus, shape of the tegular apophysis, and structure of the epigyne and vulva.

#### Description.

##### Male.

Total length 6.1 (5.6–6.25). Carapace: 3.05 (2.8–3.1) long, 2.1 (1.9–2.1) wide. Carapace length/femur IV ratio 1.07 (1.03–1.12). Habitus and pattern as in Figs 2–3.

Palp as in Figs 18–21, 23, 29–30, cymbium with distinct spines, apical part of tegular apophysis with triangular extension, embolus filiform along its entire course.

**Table:** Length of leg segments:

	femur	patella	tibia	metatarsus	tarsus	Total
I	2.3	1.05	2.0	1.85	1.25	8.45
II	2.2	1.0	1.75	1.85	1.15	7.95
III	2.15	0.9	1. 5	2.1	1.05	6.2
IV	2.85	1.05	2.25	3.2	1.45	10.8

**Table:** Spination of legs:

	femur	patella	tibia	metatarsus
I	1 or 2p+2r	1p	0 or 1p+2–2v	1p+2–2v
II	2p+2r	1p	2p+2–2v	2p+2–2v
III	2p+2r	1p+1r	2p+2r+2–2v	2p+2r+2–2v
IV	2p+2r	1p+1r	2p+2r+2–2v	2p+2r+3–2v

##### Female.

Total length 6.6 (5.7–6.6). Carapace: 2.35 (2.35–2.7) long, 1.7 (1.7–1.85) wide. Carapace length/femur IV ratio 1.18 (1.1–1.18). Habitus and pattern as in Fig. 2.

Epigyne as in Figs 35–38, septum almost triangular in shape, upper margins of windows inclined.

**Table:** Length of leg segments:

	femur	patella	tibia	metatarsus	tarsus	Total
I	1.7	0.75	1.3	1.2	0.85	5.8
II	1.6	0.7	1.1	1.1	0.8	5.3
III	1.5	0.7	0. 9	1.4	0.85	4.45
IV	2.0	0.75	1.5	2.35	1.2	7.8

**Table:** Spination of legs:

	femur	patella	tibia	metatarsus
I	2p	0	0 or 1p+3–2v	2p+2–2v
II	2p+0 or 1r	0	1p+2–2v	2p+2–2v
III	2p+1r	1p+1r	2p+1r+2–2v	2p+2r+2–2v
IV	1 or 2p+2r	1p+1r	2p+2r+2–2v	2p+2r+3–2v

#### Comments.

It seems that Schenkel (1963) placed this species in *Arctosa* due to the carapace pattern being typical for the genus (no stripes or bands). *Arctosa mongolica* was synonymized with *Xerolycosa nemoralis* by Yu & Song (1988) without examination of the female holotype. Study of the holotype and comparison with European and Siberian specimens of *Xerolycosa nemoralis* revealed clear differences in pattern, spination and copulatory organs and therefore we remove *Xerolycosa mongolica* from synonymy and establish a new combination.

#### Biology.

*Xerolycosa mongolica* females make burrows in the ground in places with sparse steppic vegetation. The burrows are relatively deep 7–10.5 cm and 4–6 mm in diameter (Logunov, personal communication). Apparently males do not construct burrows. These observations were first made by Dmitri Logunov in Tuva. Subsequently we (Koponen and Marusik) witnessed this behaviour. It is worth mentioning that *Xerolycosa mongolica* seems to be the smallest burrowing wolf spider (Logunov, personal communication).

#### Distribution.

The exact distribution of this species is unknown because the type locality is uncertain (Urga-Tsitsikar), and because of incorrect synonymisation its distribution in China is unclear. *Xerolycosa mongolica* is well documented from Tuva only.

**Figures 8–13.**
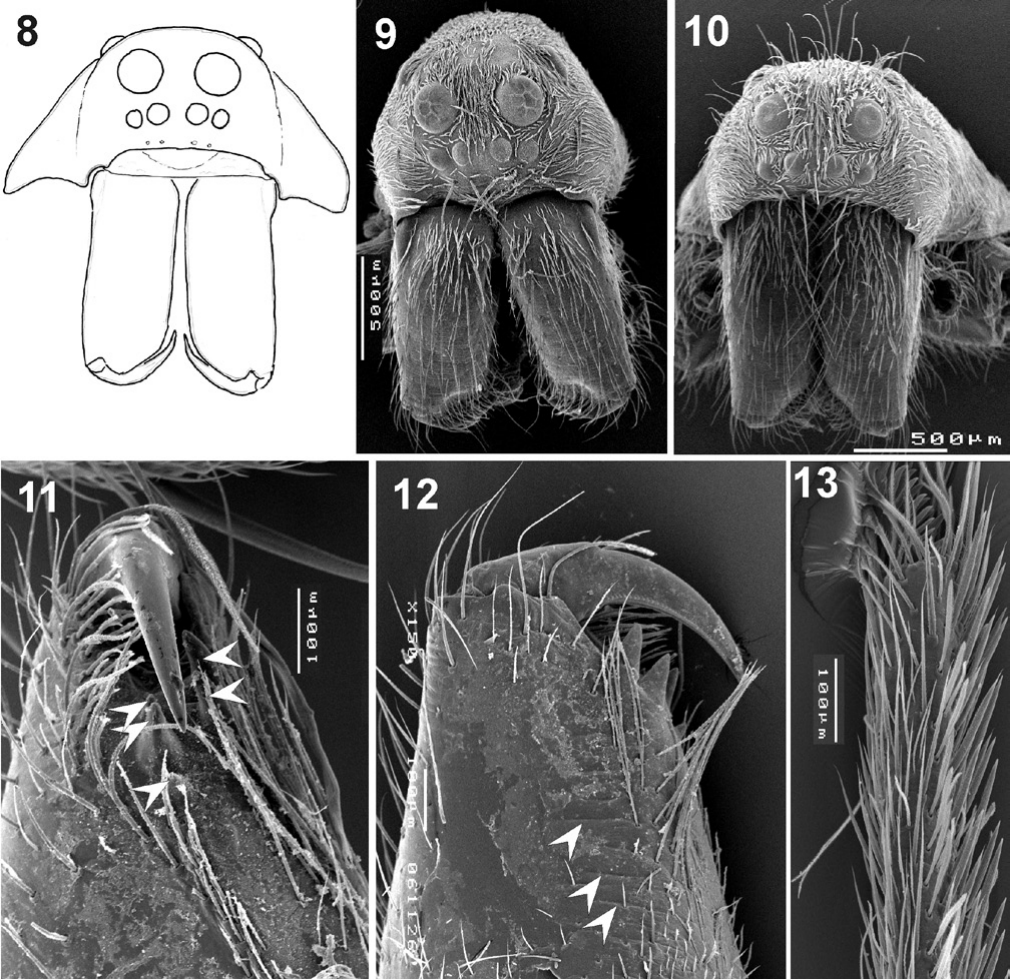
Somatic characters of *Xerolycosa nemoralis* **8–9, 11–13** and *Xerolycosa mongolica* **10** **8–10** prosoma, frontal **11–12** – chelicerae, median and inner view **13** tarsus IV, retrolateral. Arrows show cheliceral teeth and stridulatory files.

**Figures 14–21.**
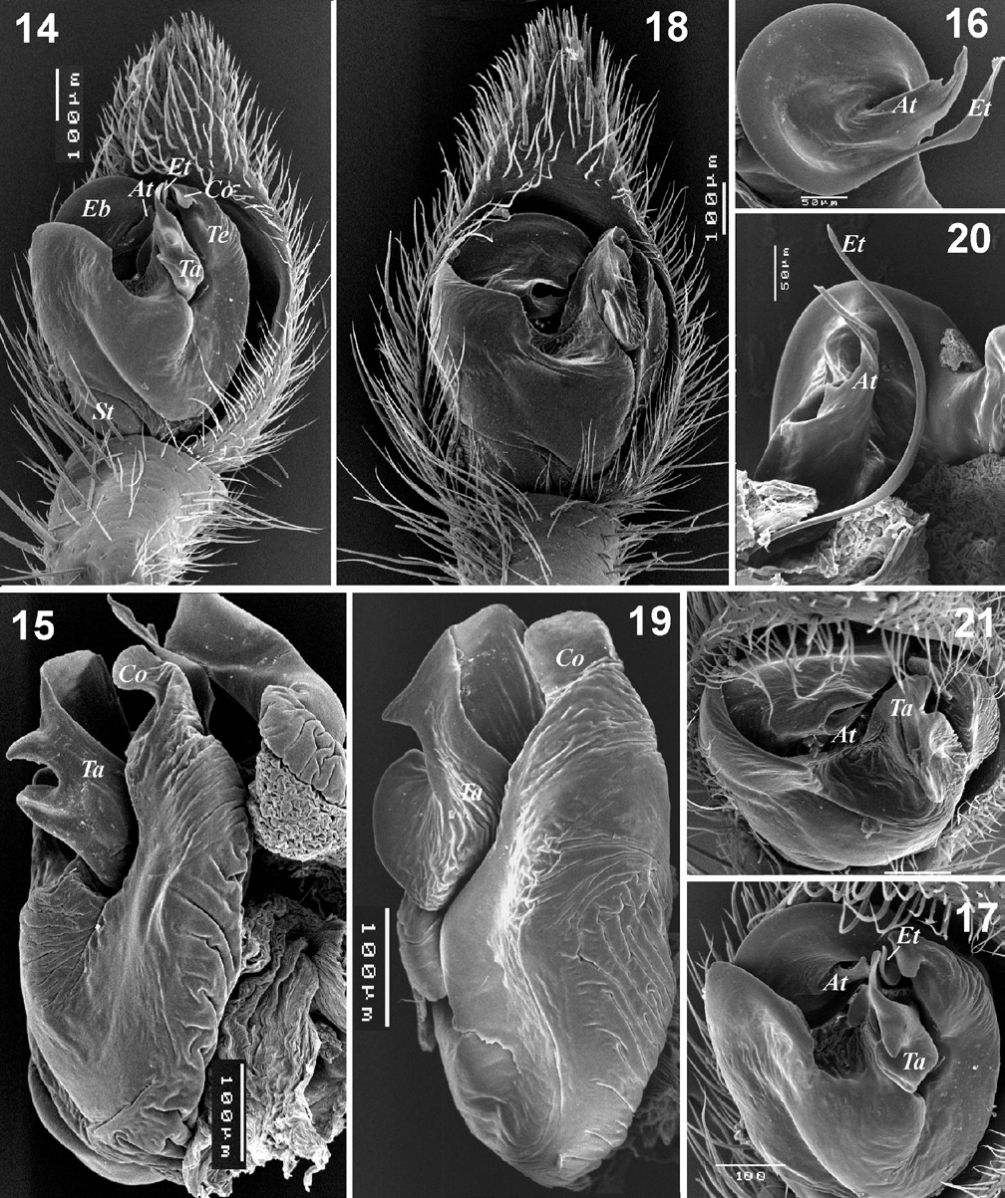
Male palp of *Xerolycosa nemoralis* **14–17** and *Xerolycosa mongolica* **18–21**. **14, 18** whole palp, ventral **15, 19** – bulbus, lateral **16, 20** embolic division, ventral and ventro-retrolateral **17, 21** whole palp, apical. Abbreviations: At– terminal apophysis; Co – conductor; Eb– base of embolus; Et – tip of embolus; St– subtegulum; Ta – tegular apophysis; Te– tegular extension.

### Xerolycosa nemoralis

(Westring, 1861)

http://species-id.net/wiki/Xerolycosa_nemoralis

Figs 4–5 8–9, 11–13 14–17 24 25–26 39–42

Lycosa nemoralis Westring, 1861: 472 (♂♀).
Xerolycosa nemoralis : Dahl 1908: 361, f. 57 (♂♀).
Tarentula flavitibia Saito, 1934: 355, pl. 13, f. 31, pl. 15, f. 84 (♀).
Xerolycosa nemoralis : Holm 1947: 23, pl. 4, f. 36–37, pl. 10, f. 25 (♂♀).
Saitocosa flavitibia : Roewer 1960: 889.
Xerolycosa nemoralis : Zyuzin 1985: 48, f. 15–16, 20–22 (♂♀).
Xerolycosa nemoralis : Roberts 1985: 140, f. 61a (♂♀).
Xerolycosa nemoralis : Roberts 1995: 222, f. (♂♀).
Xerolycosa nemoralis : Roberts 1998: 236, f. (♂♀).
Xerolycosa nemoralis : Almquist 2005: 252, f. 246a-i (♂♀).

For a complete list of references see Platnick (2011).

#### Misidentification.

*Xerolycosa nemoralis*: Yin et al. 1997: 10, f. 3a-d (♀), refer to a species with unclear generic affinities.

#### Material examined.

**FINLAND** (selected records from different parts of the range): 18♂♀ (ZMUT), Vuolijoki, Vuottolahti, Lapinniemi (64°13'N, 27°20'E), 16.07.1972 (P.T. Lehtinen); 1♂ (ZMUT), Hammarland Sålis (60°15'N, 19°44'E), dry forest, 26.06.-06.08.1971 (P.T. Lehtinen); 5♂ 4♀ (ZMUT), Turku Kärsämäki (60°30'N, 22°15'E), forest, 24.04.-04.08.1972 (I. Oksala); 12♂♀ (ZMUT), Harjavalta, Sport center (61°17'N, 22°10'E), pine forest, 09.07.-09.08.1992 (S. Koponen). **RUSSIA:** Adygeya:2 ♀ (TNU-2718/18), Caucasian State Reserve, env. kordon Guzeripl (44°00'N, 40°08'E, ~ 670 m), *Abies* & *Fagus* wood, 13–17.08.2009 (M.M. Kovblyuk); 2 ♀ (TNU-2719/3), Caucasian State Reserve, env. kordon Guzeripl (44°00'N, 40°08'E, ~ 670 m), *Abies* & *Fagus* wood, pitfalls, 16-23.08.2009 (M.M. Kovblyuk). Krasnoyarsk Prov.**:** 1♀ (IBPN), West Sayany Mts., Oiskiy Mt. Range, Buiba Riv., 52°47'N, 93°18'E, 1230 m, among stones, 20-21.06.1995 (Yu.M. Marusik). Sakhalin Island**:** 4♂ 4♀ 13 juv. (IBPN), SE part, Tsapko Vill. env., Zhdanko Mt. Range, 48°06.051'N, 142°31.557'E, h 220m, foothill of Vladimirovka Mt., stony debris along temporary creek, 1.08.2001 (Y.M.Marusik ); 5♂ 2♀ (IBPN), SE part, env. of Starodubskoye Vil., Naiba River mouth part, 47°24.992'N, 142°45.384'E, 23.07.2001 (Y.M.Marusik); 4♂ 5♀ (IBPN), SW part, Krilyon Peninsula, W shore, ca 5 km S of Shebunino Vil., Kitosia River mouth, 36°22.536'N, 141°52.562'E, 14-15.08.2001 (Y.M.Marusik). Magadan Area**:** 1♂ (IBPN), ca. 30 km N of Magadan, Dukcha River Valley, gravely bank, June 1995 (Y.M.Marusik). Kamchatka Province**:** 1♂ (IBPN), Kamchatka Peninsula, 10–12 km N of Paratunka Vil., Yelizovo Forestry, 53.050°N, 158.225°E, 15-28.07.2004 (A.S. Ryabukhin). No precise data: numerous males and females have been collected on five Kuril Islands**:** Kunashir, Iturup, Urup, Chirpoi and Paramushir.

#### Diagnosis.

The species differs distinctly from *Xerolycosa mongolica* by the pattern of its carapace, having longitudinal bands and stripes, and by having the anterior median eyes situated more closely together (less than one diameter of AME, more than one diameter in *Xerolycosa mongolica*). From *Xerolycosa miniata*, males can be distinguished by their longer seminal duct, rounded embolus, the sharply pointed process of the tegular apophysis and by the proportions of the epigyne (windows wider than high, whereas in *Xerolycosa miniata* they are higher than wide). *Xerolycosa nemoralis* females possess 2 retrolateral spines on femur I whereas the other species have only 2 prolateral spines.

#### Description.

##### Male.

Total length 6.0 (5.5–6.8). Carapace: 2.9 (2.75–2.9) long, 2.0 (1.9–2.0) wide. Carapace length/femur IV ratio 1.23 (1.12–1.23). Habitus and pattern as in Fig. 4.

Palp as in Figs 14–17, 24–26, tip of cymbium with poorly developed spines, upper part of tegular apophysis with bill-shaped extension, embolus relatively thick, free part (=embolus proper) bent, tip modified.

**Table:** Length of leg segments:

	femur	patella	tibia	metatarsus	tarsus	Total
I	1.85	0.85	1.5	1.55	1.05	6.8
II	1.85	0.85	1.35	1.4	1.1	6.55
III	1.7	0.8	1.25	1.75	1.05	6.55
IV	2.35	1.0	1.8	2.8	1.35	9.3

**Table:** Spination of legs:

	femur	patella	tibia	metatarsus
I	2p+2r	1p+1r	1p+2r+3–2v	2p+1r+2–2v
II	2p+2r	1p+1r	2p+2r+2–2v	2p+1r+2–2v
III	2p+2r	1p+1r	2p+2r+2–2v	2p+2r+2–2v
IV	2p+1r	1p+1r	2p+2r+2–2v	2p+2r+3–2v

##### Female

Total length 6.7 (6.4–7.1). Carapace: 3.25 (2.8–3.35) long, 2.25 (2.0–2.4) wide. Carapace length/femur IV ratio 1.18 (1.14–1.18). Habitus and pattern as in Fig. 5.

Epigyne as in Figs 39–42, windows wider than high, septum with rounded sides.

**Table:** Length of leg segments:

	femur	patella	tibia	metatarsus	tarsus	Total
I	2.25	1.0	1.75	1.7	1.3	8
II	2.15	0.95	1.65	1.65	1.3	7.7
III	2.15	0.9	1. 6	2.0	1.25	6.3
IV	2.75	1.05	2.2	3.25	1.5	10.75

**Table:** Spination of legs:

	femur	patella	tibia	metatarsus
I	2p+2r	1p	1p+3–2v	2p+2–2v
II	2p+2r	1p	1p+2–2v	2p+2–2v
III	2p+2r	1p+1r	2p+2r+2–2v	2p+2r+2–2v
IV	2p+1r	1p+1r	2p+1r+2–2v	2p+2r+3–2v

#### Comments.

Judging from the figures, the record of *Xerolycosa nemoralis* by Yin et al. (1997: f. 3a-d) from China refers to another species and even a different genus.

#### Distribution.

*Xerolycosa nemoralis* has a trans-Palaearctic boreo-nemoral range (Marusik et al. 2000) and occurs from the Iberian Peninsula to Kamchatka and the North Kuril Islands, north to the Polar Circle in Lapland and to central Yakutia, south to Azerbaijan and Honshu.

**Figures 22–24.**
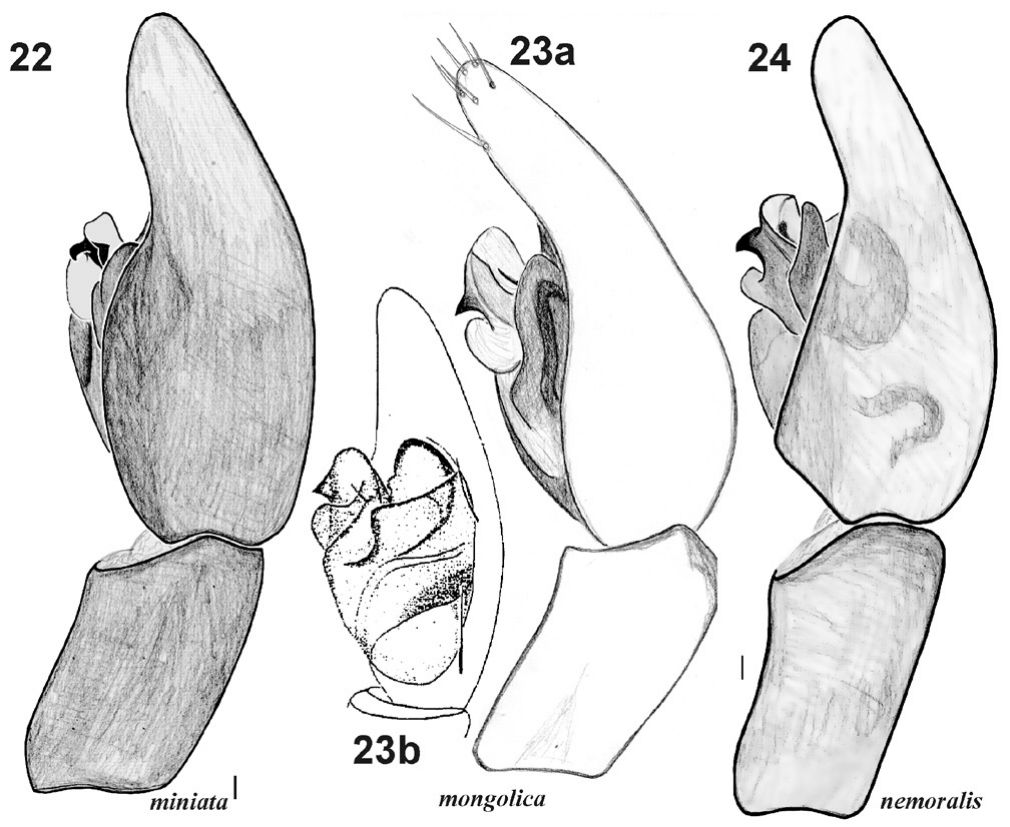
Male palp of *Xerolycosa miniata* **22** *Xerolycosa mongolica* **23** and *Xerolycosa nemoralis* **24** **22, 23a, 24** retrolateral **23b** prolateral. **23b** after Chen et al. (1998). (scale bar 0.1 mm).

**Figures 25–30.**
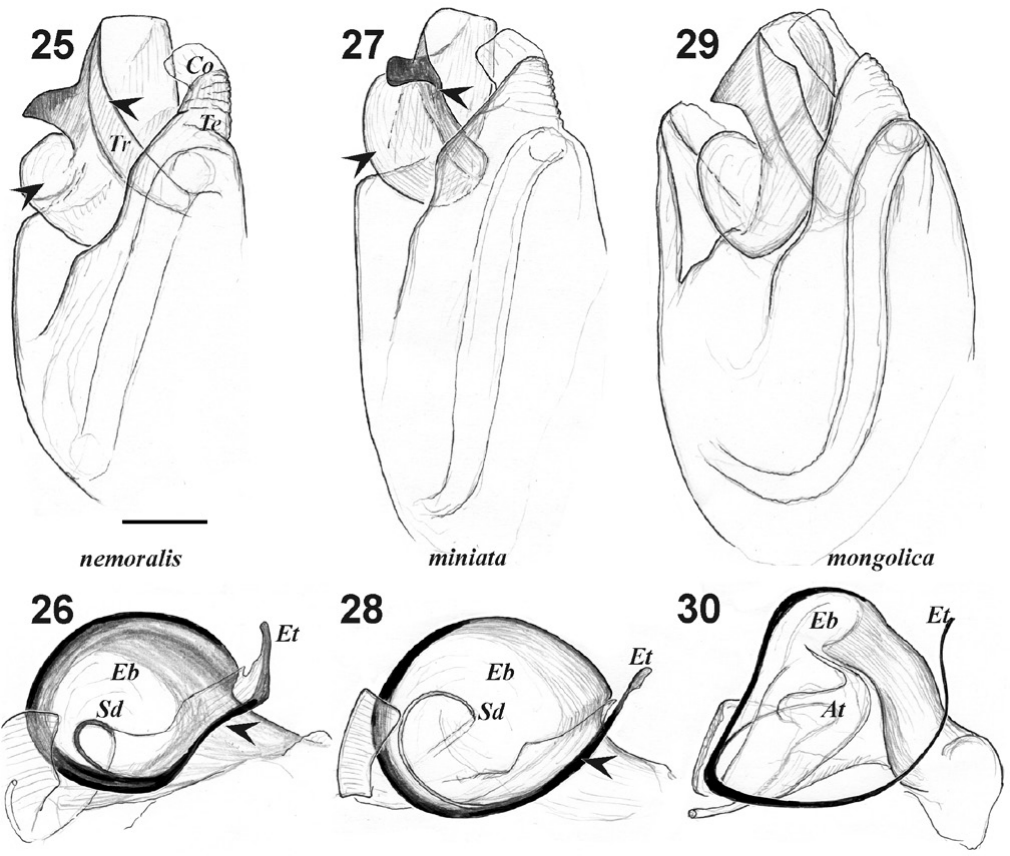
Male palp of *Xerolycosa nemoralis* **25–26** *Xerolycosa miniata* **27–28** and *Xerolycosa mongolica* **29–30** **25, 27, 29** bulbus, retrolateral **26, 28, 30** embolic division, ventral. Arrows indicate differences between *Xerolycosa miniata* and *Xerolycosa nemoralis.* Abbreviations: At– terminal apophysis; Co – conductor; Eb –base of embolus; Et – tip of embolus; Sd – seminal duct.

**Figures 31–42.**
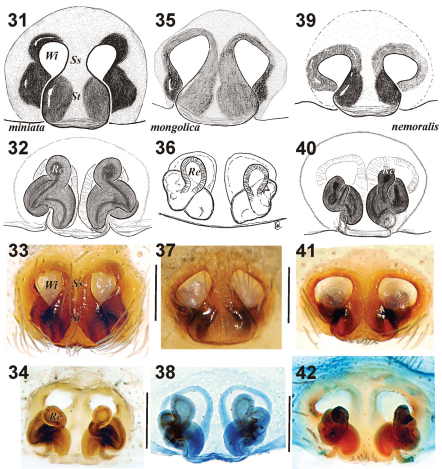
Epigyne of *Xerolycosa miniata* **31–34** *Xerolycosa mongolica* **35–38** and *Xerolycosa nemoralis* **39–42** **31, 33, 35, 37, 39, 41** ventral **32, 36, 40, 34, 38, 42** dorsal. (**36** & **38** holotype). Abbreviations: Re– receptaculum; Se– septum; Ss *–* septal stem;Wi – window of epigyne.

## Supplementary Material

XML Treatment for Xerolycosa

XML Treatment for Xerolycosa miniata

XML Treatment for Xerolycosa mongolica

XML Treatment for Xerolycosa nemoralis
